# Healthcare practitioners' acceptance of using telehealth in the Kingdom of Saudi Arabia: an application of the unified theory of acceptance and use of technology model

**DOI:** 10.3389/fdgth.2025.1659997

**Published:** 2025-11-04

**Authors:** Abdullah A. AlMojaibel, Abdulelah Aldhahir, Khalid Aldilaijan, Rayyan Almusally, Marah AlAtrash, Mohammad A. Alkhofi, Saeed M. Alghamdi, Yousef Alqurashi, Mohammed Alsubaiei, Khalid AlHarkan, Jithin K. Sreedharan, Shoug Al Humoud

**Affiliations:** ^1^ Department of Respiratory Care, Imam Abdulrahman Bin Faisal University, Dammam, Saudi Arabia; ^2^ Department of Nursing, College of Nursing and Health Sciences, Jazan University, Jazan, Saudi Arabia; ^3^ Department of Otorhinolaryngology, King Fahd Military Medical Complex, Dhahran, Saudi Arabia; ^4^ Internal Medicine Department, King Fahd Hospital of the University, Imam Abdulrahman Bin Faisal University, Khobar, Saudi Arabia; ^5^ Virtual Healthcare Administration, Eastern Health Cluster, Dammam, Saudi Arabia; ^6^ Department of Pediatrics, King Fahad University Hospital, Imam Abdulrahman Bin Faisal University, Al Khobar, Saudi Arabia; ^7^ Clinical Technology Department, Respiratory Care Program, College of Applied Medical Sciences, Umm Al-Qura University, Makkah, Saudi Arabia; ^8^ Department of Physical Therapy, Faculty of Applied Medical Sciences, Imam Abdulrahman bin Faisal University, Dammam, Saudi Arabia; ^9^ Department of Family and Community Medicine, College of Medicine, Imam Abdulrahman Bin Faisal University, Dammam, Saudi Arabia; ^10^ Department of Respiratory Therapy, College of Rehabilitation Sciences, University of Manitoba, Winnipeg, MB, Canada

**Keywords:** telehealth, telemedicine, healthcare practitioners, acceptance, intention, UTAUT, Kingdom of Saudi Arabia

## Abstract

**Introduction:**

Telehealth offers several advantages over traditional in-person clinic visits. Despite its potential benefits, some barriers affect the optimal use of telehealth. Understanding healthcare practitioners' (HCPs) acceptance of telehealth is essential to ensure the successful, high-quality, and safe implementation of telehealth programs. However, a comprehensive, theory-driven understanding of the factors influencing HCPs' acceptance of telehealth in the Kingdom of Saudi Arabia (KSA) is lacking, hindering the development of effective implementation strategies. Therefore, this study aimed to measure telehealth acceptance among HCPs in the KSA and to identify the key predictors of their intention to use it, with a specific focus on constructs derived from the Unified Theory of Acceptance and Use of Technology (UTAUT).

**Methods:**

This study was conducted from June 2024 to January 2025. HCPs working in the KSA were included. The survey was grounded in the Unified Theory of Acceptance and Use of Technology (UTAUT) and consisted of four constructs: performance expectancy (PE), effort expectancy (EE), social influence (SI), and facilitating conditions (FC). In addition to behavioral intention (BI) as the dependent variable. The data analysis included performing descriptive analysis for the sociodemographic variables and multivariate logistic regression analysis.

**Results:**

A total of 1,051 HCPs completed the survey. The analysis indicated that 97.8% of respondents expressed a positive intention to use telehealth in the future. Performance expectancy (PE) emerged as a significant predictor of the intention to use telehealth [adjusted odds ratio (AOR) = 4.45, *p* < 0.05], as did social influence (SI) (AOR = 19.25.2, *p* < 0.01). Furthermore, employment in military or private hospitals was associated with a significantly lower likelihood of intending to use telehealth (AOR = 0.05, *p* < 0.05 and AOR = 0.07, *p* < 0.05, respectively).

**Conclusion:**

This study represents the first large-scale, theory-driven investigation of telehealth acceptance among healthcare practitioners (HCPs) across all regions of the KSA. The findings underscore the critical role of performance expectancy (PE) and social influence (SI) as significant predictors of HCPs' intention to adopt telehealth services. These insights provide valuable direction for policymakers and healthcare leaders, emphasizing the importance of fostering supportive professional environments and highlighting perceived benefits to enhance telehealth adoption. To address concerns related to effort expectancy and the facilitating conditions, telehealth developers should prioritize user-friendly designs and provide accessible and responsive IT support for users.

## 1 Introduction

High-quality healthcare services and improved accessibility are key indicators of a society's development (1). The global burden of chronic medical conditions among adults continues to rise, contributing to increased healthcare costs and elevated mortality rates, particularly among those with multiple comorbidities (2, 3). Adults with multiple chronic medical conditions have higher healthcare costs and an increased risk of death (4). Managing the growing number of patients with chronic conditions is challenging for healthcare systems, especially in rural areas (5, 6). Thus, governments and healthcare agencies are trying new ways to provide efficient and high-quality healthcare services (7). The emerging telecommunication technology can potentially solve the healthcare system's issues, including increased referrals and limited access to care (8–10). Telehealth is defined as providing or receiving healthcare services through different channels of telecommunication technologies, including smartphones, computers, and the internet (11). Telehealth consists of different modalities that can be used for various medical conditions and patient populations for monitoring, diagnosis, treatment, prevention, continuous education, and administrative functions (1, 12, 13).

Telehealth offers several advantages over traditional in-person clinic visits, including improved access to healthcare services, reduced costs, and enhanced quality of care (14–23). Despite its potential benefits, some barriers affect the optimal use of telehealth, such as concerns about ease of use, lack of physical examination, and limited in-depth communication (24). Healthcare practitioners (HCPs) may also perceive telehealth as challenging to learn and implement, both for themselves and their patients (17, 22).

Telehealth was first introduced in the Kingdom of Saudi Arabia (KSA) by the King Faisal Specialist Hospital and Research Center (KFSHRC) in Al-Riyadh, where it was primarily used for consultations between HCPs (25). The broader adoption of telehealth in the KSA began in 2018 as part of an initiative from the Ministry of Health (MOH) and the private healthcare sector to implement multiple telehealth programs and applications (26). These programs and applications have since been utilized for medical teleconsultations, e-prescriptions, and general support services for HCPs (27, 28).

Research studies conducted in various countries have assessed telehealth acceptance among HCPs and identified key factors influencing its adoption (23, 24, 29, 30). Only a few studies have measured the acceptance of using telehealth among HCPs in the KSA. These studies found that perceived usefulness and ease of use were positive predictors of telehealth adoption (22, 31). Telehealth can potentially increase the effectiveness of healthcare services, improve the quality of care, provide faster services, and help users save time and money (32–34). However, barriers to using telehealth were also explored in the context of the KSA. These include the limited availability of telehealth programs, network connectivity issues (31), inability to conduct physical examinations (32), and concerns that using telehealth could reduce the effectiveness of patient care (33). Understanding HCPs' acceptance of telehealth is essential to ensure the successful, high-quality, and safe implementation of telehealth programs (35). Their acceptance is also key to ensuring the sustainability of these programs (36). However, a lack of acceptance was found to be a barrier to telehealth implementation (37). It is important to note that recent studies that measured telehealth acceptance among HCPs in the KSA have several limitations, including a small sample size (32, 33) and a lack of a theoretical framework (31–34).

Our literature review identified multiple models and theoretical frameworks that explain users' intentions to utilize new technologies, such as telehealth. These models include Theory of Reasoned Action (TRA) and Theory of Planned Behavior (TPB), the Health Belief Model (HBM), Diffusion of Innovations theory (DOI), and the Technology Acceptance Model (TAM). The TRA suggests that intention to use technology is a product of attitude and social norms. In addition to the two factors, TPB has an additional construct (the perception of a capacity to control) (38). HBM is a widely applied model for comprehending the behavioral intentions for certain health programs or behaviors (38). According to the HBM, intention to use new technology is determined by the perceptions of susceptibility, severity, benefits, barriers, cues to action, motivating factors, and self-efficacy (39). DOI model is usually utilized in explaining the adoption process of new innovations (39). According to the DOI model, the adoption of a new behavior is influenced by the following constructs: relative advantages, compatibility, complexity, trialability, and observability (38). Moreover, the TAM model was developed by Fred D. Davis to help in predicting users' intention to use new technologies by two factors: perceived usefulness (PU) and perceived ease of use (PEOU) (40). In 2003, Venkatesh and Davis developed the Unified Theory of Acceptance and Use of Technology (UTAUT) (41), as a comprehensive model to predict technology acceptance. The model comprises four core constructs: performance expectancy (PE), effort expectancy (EE), social influence (SI), and facilitating conditions (FC) (41). A review of recent literature indicates that the UTAUT model is extensively employed in telehealth acceptance research across diverse healthcare disciplines internationaly, thereby affirming its applicability and construct validity (21, 42–44). Moreover, the UTAUT demonstrate superior explanatory power compared to the other existing technology acceptance models (42). Research applying the UTAUT model to analyze telehealth acceptance in the KSA remains scarce, with only one identified study employing this framework. Alaboudi et al. (2016) integrated the UTAUT constructs with additional elements derived from other behavioral theories to guide their qualitative research framework (45).

**Figure 1:**
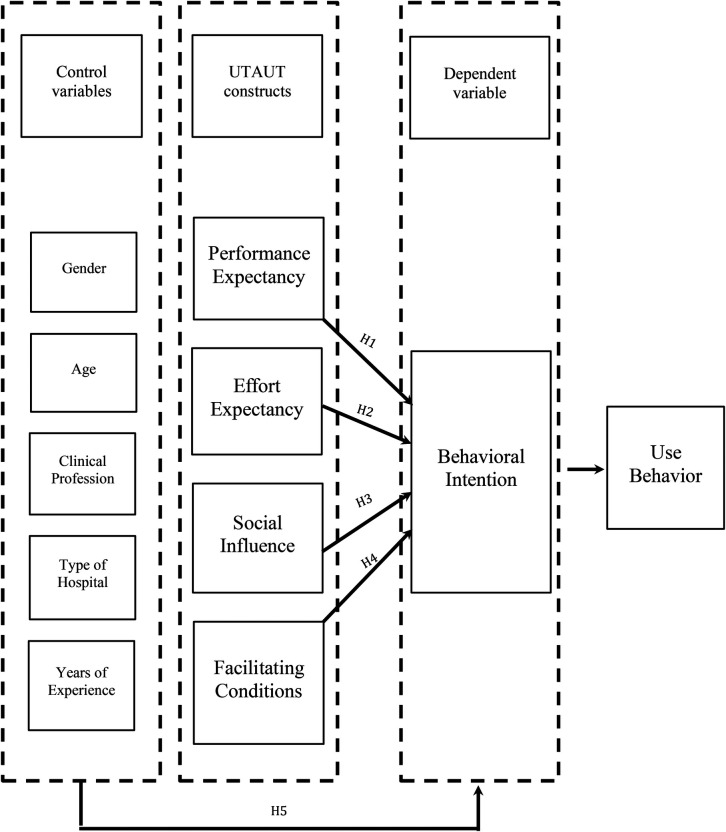
Research model based on the unified theory of acceptance and use of technology (UTAUT).

The UTAUT model was developed through an empirical synthesis of proceeding technology acceptance models, integrating their most significant constructs into a unified model (41). The UTAUT model is built upon four primary constructs: PE, EE, SI, and FC. See Figure 1. PE is defined as “the degree to which an individual believes that using the system will help him or her to attain gains in job performance” (41). In the context of telehealth, PE is the degree to which a user believes that using telehealth will be associated with clinical and other benefits. PE is conceptually aligned with PU, benefits, and relative advantages from other health behavior models. The second core construct of the UTAUT is EE. It is defined as “the degree of ease associated with the use of the system” (37). EE reflects the users' perception of the ease of using telehealth. EE in the UTAUT is conceptually equivalent to the PEOU from the TAM. The third construct of the UTAUT is SI. SI is defined as “the degree to which an individual perceives that important others believe he or she should use the new system” (41). As a concept, SI pertains to the influence of colleagues or supervisors who are perceived as encouraging telehealth adoption and it is comparable to the social norms construct from the TRA. FC is defined as “the degree to which an individual believes that an organizational or technical infrastructure exists to support the use of the system” (37). In the context of telehealth, FC denotes the availability of the necessary organizational and technical support to use telehealth services. The inclusion of the FC construct represents a distinctive aspect of the UTAUT, as this construct was not explicitly addressed in other health behavior frameworks. Additionally, HCPs' sociodemographic characteristics such as gender, age, clinical profession, hospital type, and years of experience were proposed as moderating variables to the acceptance of telehealth.

Within telehealth research, common theoretical frameworks have been applied to investigate telehealth acceptance including the TPB (46), the HBM (47–49), the DOI (50, 51), and the TAM (17, 52–54). These frameworks typically emphasize individual attitudes or perceptions but may not fully capture the combined social, organizational, and infrastructural influences that shape telehealth acceptance in real-world clinical settings. In the Saudi context, current studies on telehealth acceptance have been limited by small sample sizes, a lack of theoretical grounding, and narrow focus on perceived usefulness and ease of use. These gaps highlight the need for a more comprehensive and integrative framework. The UTAUT was therefore selected for this study due to its capacity to capture the complexity of factors influencing healthcare practitioners' acceptance of telehealth in the KSA.

Based on the UTAUT theoretical framework, the study proposed the following hypotheses:

*H1: Performance expectancy (PE) is a significant positive predictor of telehealth acceptance.*
*H2: Effort expectancy (EE) is a significant positive predictor of telehealth acceptance.*
*H3: Social influence (SI) is a significant positive predictor of telehealth acceptance.*
*H4: Facilitating conditions (FC) is a significant positive predictor of telehealth acceptance.*
*H5: Sociodemographic variables of HCPs are significant positive predictors of telehealth acceptance.*

Despite the rapid expansion of telehealth services in the KSA (28), its acceptance remains under-investigated. Therefore, to fill the gap and to address the limitations of the recent studies, the present study was designed based on a well-established theoretical framework to investigate telehealth acceptance among HCPs in the KSA. It also sought to examine the associations between sociodemographic variables such as gender, age, clinical profession, type of hospital, years of experience, and the intention to use telehealth. Specifically, the study addressed the following research questions: (1) What factors influence the acceptance of telehealth among HCPs in the KSA? (2) Which sociodemographic variables are associated with telehealth acceptance among HCPs in the KSA?

Answering these questions will help bridge the existing gap in the literature concerning the role of perceived usefulness, perceived ease of use, social influence, facilitating conditions, and sociodemographic factors in shaping HCPs' acceptance of telehealth within the KSA. The findings of this study will provide valuable insights for healthcare decision-makers aiming to implement or expand telehealth services. By understanding the factors that influence acceptance, healthcare leaders can tailor future telehealth initiatives to better meet HCPs' needs and enhance program uptake and sustainability.

## 2 Methods

### 2.1 Study design

This survey-based study was conducted from June 2024 to January 2025. HCPs aged 18 years or older working in the KSA were included. Ethical approval for the study was obtained from the Institutional Review Board of Imam Abdulrahman Bin Faisal University. All the potential participants were informed about the aim and purpose of the study, that their participation was voluntary, and they were informed that their responses would remain anonymous. Informed consent was obtained from the participants through the following statement: “If you are a healthcare practitioner in the KSA and consent to participate in this survey, please proceed to the next page to start the survey”. Only those who answered “yes” were allowed to proceed to the questionnaire.

A chain-referral, convenient sampling approach was utilized to recruit HCPs for this study. The survey was administered online using the QuestionPro^™^ software (https://www.questionpro.com). The survey link was distributed through social media platforms and sent directly to HCPs via emails and direct messages. To enhance national reach and improve the representativeness of the sample, the data collection team shared the survey link with colleagues across various healthcare sectors and regions of the KSA. To prevent duplicate or unauthentic responses, survey settings were configured to restrict multiple submissions from the same individual. Additionally, the research team made targeted efforts to engage underrepresented groups by sending multiple rounds of follow-up reminders, aiming to reduce non-response bias in certain demographic or geographic segments.

### 2.2 Sample size estimation

The required sample size was calculated using the formula: *n* = z² * p * q/e^2^, where *n* is the required sample size, *p* is the estimated prevalence (58% of HCPs' satisfaction with telehealth applications) (28), q = 1−p, z = 1.96 (corresponding to a 95% confidence level), and e = 0.05 (margin of error). Substituting the values: *n* = (1.96)^2^ × 0.58 × (1−0.58)/(0.05)^2^ = 384. Thus, the minimum required sample size was estimated to be 384 participants. According to the most recent statistics from the Ministry of Health, there were approximately 550,000 HCPs in Saudi Arabia in 2023 (38). Given that the survey was conducted online, the sample size was increased to 1,000 participants to enhance statistical power and improve generalizability.

### 2.3 Data collection procedure

The questionnaire used in this study consisted of two sections. The first section collected sociodemographic data, including, age, clinical profession, hospital type, and years of telehealth experience. Age was categorized as follows: 20–29 years, 30–39 years, 40–49 years, 50–59 years, and 60 years or older. Clinical professions were classified as physicians, nurses, dentists, pharmacists, other healthcare specialists (e.g., respiratory therapists, physiotherapists, clinical nutritionists), and technicians in allied medical sciences. Also, participants were asked about the type of hospital they work in. This variable was categorized into primary care, tertiary hospital, university hospital, military hospital, private hospital, or others. Years of telehealth experience was divided into five categories: never used telehealth, 1 year or less, 2–4 years, and 5 years or more.

The second section of the questionnaire employed the Saudi Telehealth Acceptance Scale, a validated, theory-based instrument developed by Almojaibel (2024) (55). Grounded in the Unified Theory of Acceptance and Use of Technology (UTAUT) (43), the scale includes four core constructs: performance expectancy (PE), effort expectancy (EE), social influence (SI), and facilitating conditions (FC), with behavioral intention (BI) serving as the dependent variable. All items were rated on a 5-point Likert scale, ranging from 1 (strongly disagree) to 5 (strongly agree). A participant was considered to agree with an item if their score exceeded 50% of the total possible score. Completion of all items was mandatory for survey submission.

Several control variables were included in the analysis to enhance the accuracy of the conclusions. Based on previous studies on telehealth acceptance, relevant sociodemographic factors—such as gender, age, clinical profession, hospital type, and years of clinical experience—were identified as potential influencers. These variables were controlled for in the statistical analysis to minimize their confounding effects on telehealth acceptance and the intention to use telehealth.

### 2.4 Statistical analysis

Data analysis was conducted using SPSS version 26.0 (IBM Corporation, New York, NY, USA). Descriptive statistics for sociodemographic variables were presented as frequencies and percentages. Bivariate logistic regression was used to examine the associations between participants' sociodemographic characteristics and each of the UTAUT constructs, including behavioral intention (BI) to using telehealth. Multivariate logistic regression analysis was conducted to estimate adjusted odds ratios (AORs) with corresponding 95% CIs and exact *p*-values. The analysis included all fully completed survey responses; therefore, no imputation for missing data was necessary. A *p*-value of < 0.05 was considered statistically significant.

## 3 Results

### 3.1 Sociodemographic information

Out of 1,409 participants, 1,051 (74.6%) completed the survey. Among the respondents, 73% were male and 27% were female. Approximately 46% of participants were aged between 20 and 29 years. Detailed sociodemographic characteristics are presented in Table 1.

**Table 1:** Sociodemographics of the study population.

Sociodemographics	Participants (*n* = 1,051)	Percent
Age (years)		
20–29	479	45.6
30–39	348	33.1
40–49	165	15.7
50–59	45	4.3
60 or older	14	1.3
Clinical profession		
Physician	347	33.0
Nurse	307	29.2
Dentist	49	4.7
Pharmacist	89	8.5
Other healthcare specialist	206	19.6
Technician in allied health sciences	24	2.3
Other	29	2.8
Type of hospital		
Primary care	328	31.2
Tertiary hospital	140	13.3
University hospital	151	14.4
Military hospital	122	11.6
Private hospital	253	24.1
Other	57	5.4
Experience of using telehealth		
I never used	543	51.7
1 year or less	202	19.2
2- 4 years	220	20.9
5 years or more	86	8.2

### 3.2 Reliability analysis

Internal consistency reliability was assessed for each subscale using Cronbach's alpha. An acceptable alpha value is typically between 0.70 and 0.80 (56). The results demonstrated strong internal consistency for all subscales: performance expectancy (PE) and effort expectancy (EE) each had a Cronbach's alpha of 0.89, social influence (SI) had 0.86, and facilitating conditions (FC) had 0.84. No improvements in Cronbach's alpha were observed upon the deletion of any items; therefore, all items were retained in the final versions of the subscales.

### 3.3 Predicting behavior intention to use telehealth based on the UTAUT constructs and the sociodemographic variables

The analysis revealed that 97.8% of healthcare practitioners (HCPs) expressed a positive intention to utilize telehealth in the future (Table 2). The findings supported Hypothesis 1, demonstrating that performance expectancy (PE) was a significant predictor of the intention to use telehealth (AOR = 4.45, *p* < 0.05). HCPs reported that telehealth would enable them to perform clinical tasks more efficiently, save time, reduce healthcare costs, and improve the overall quality of care (Table 3). Additionally, the data supported Hypothesis 3, showing that social influence (SI) was a strong predictor of telehealth acceptance (AOR = 19.25, *p* < 0.01) (Table 4). These findings indicate that perceived clinical and operational gains, alongside normative pressures from both peers and administrative leadership are primary determinants of telehealth acceptance in the KSA. Effort expectancy (EE) was not a statistically significant predictor of the intention to use telehealth (AOR = 1.92, *p* = 0.38); therefore, Hypothesis 2 was not supported. Notably, participants expressed concerns about the anticipated difficulty of resolving technical issues related to telehealth use (Table 5). Similarly, facilitating conditions (FC) did not significantly predict the intention to use telehealth (AOR = 2.56, *p* = 0.13), and thus, Hypothesis 4 was not supported. HCPs reported uncertainty regarding the availability of adequate technical support to address telehealth-related challenges (Table 6). Regarding workplace context, hospital type was the only sociodemographic factor associated with BI after adjustment; HCPs working in military or private hospitals reported significantly lower intention to use telehealth than those in primary care centers. In contrast, no significant associations were found between telehealth acceptance and the other sociodemographic variables (age, profession, and years of experience with telehealth) after adjustment. This pattern suggests that organizational policies and on-the-ground implementation environments may matter more than individual attributes for telehealth acceptance among HCPs in the KSA.

**Table 2:** Behavioral intention (BI): acceptance of telehealth among the study population.

Behavioral Intention (BI)	Mean ± SD
I am positive toward using the telehealth.	4.3 ± 0.8
I will use the telehealth when it becomes available.	4.3 ± 0.8
I am willing to use telehealth to provide care services.	4.3 ± 0.8
I will use the telehealth to provide health care services as often as needed.	4.3 ± 0.8
Behavioral intention (BI) Total Score	
Positive (Score >10)	17.3 ± 2.8
Negative (Score ≤10)	

**Table 3:** Logistic regression analysis for the performance expectancy (PE) items that could influence the intention to use telehealth.

PE items	Intention to use telehealth	
	Adjusted OR	*p*-value
Telehealth will allow me to accomplish my clinical tasks more quickly.		
Strongly disagree	Ref	
Disagree	4.42 (0.25–77.07)	0.309
Neutral	7.01 (0.72–68.33)	0.093
Agree	77.96 (4.85–1,251.75)	0.002*
Strongly agree	39.67 (2.32–679.58)	0.011*
Telehealth will save me time.		
Strongly disagree	Ref	
Disagree	0.40 (0.2–6.32)	0.516
Neutral	9.97 (0.97–102.66)	0.053
Agree	19.06 (1.74–208.78)	0.016*
Strongly agree	5.75 (0.45–73.39)	0.178
Telehealth will increase the quality of the health care services.		
Strongly disagree	Ref	
Disagree	5.07 (0.31–82.66)	0.254
Neutral	2.30 (0.25–21.03)	0.462
Agree	3.34 (0.33–33.51)	0.306
Strongly agree	59.20 (1.76–1,994.57)	0.023*
Telehealth will decrease the cost of the health care services.		
Strongly disagree	Ref	
Disagree	0.40 (0.01–32.81)	0.685
Neutral	0.50 (0.01–21.32)	0.715
Agree	0.04 (0.001–2.16)	0.117
Strongly agree	0.02 (0.00–1.28)	0.066
Telehealth will facilitate the monitoring of the disease.		
Strongly disagree	Ref	
Disagree	0.33 (0.02–6.41)	0.465
Neutral	3.81 (0.42–34.41)	0.233
Agree	1.58 (0.14–18.07)	0.712
Strongly agree	1.52 (0.11–21.17)	0.755
Telehealth is useful for the health care system.		
Strongly disagree	Ref	
Disagree	18.29 (0.51–651.15)	0.111
Neutral	36.07 (2.84–458.18)	0.006*
Agree	30.21 (2.23–409.71)	0.010*
Strongly agree	264.51 (3.95–17,712.99)	0.009*
Telehealth will improve the relationship between the health care provider and the patient.		
Strongly disagree	Ref	
Disagree	2.27 (0.14–37.62)	0.567
Neutral	0.71 (0.6–7.99)	0.785
Agree	1.25 (0.11–14.50)	0.861
Strongly agree	9.11 (0.15–561.48)	0.293
Performance expectancy (PE)		
Disagree (<18 score)	Ref	
Agree (≥18 score)	4.45 (1.07–18.39)	0.039*

* *p*-value significant.

**Table 4:** Logistic regression analysis for the social influence (SI) items that could influence the intention to use telehealth.

SI items	Intention to use telehealth	
	Adjusted OR	*p*-value
Most people who are important to me think I should use telehealth.		
Strongly disagree	Ref	
Disagree	2.94 (0.23–37.89)	0.407
Neutral	2.17 (0.31–15.16)	0.434
Agree	15.13 (0.93–247.27)	0.057
Strongly agree	0.77 (0.10–5.79)	0.799
People whose opinions I value would prefer me to use telehealth.		
Strongly disagree	Ref	
Disagree	0.65 (0.58–7.14)	0.721
Neutral	2.19 (0.26–18.42)	0.472
Agree	1.33 (0.11–16.06)	0.822
Strongly agree	-	0.993
The management would motivate me to use telehealth.		
Strongly disagree	Ref	
Disagree	0.33 (0.03–3.37)	0.351
Neutral	6.56 (1.01–42.61)	0.049*
Agree	24.90 (2.83–218.94)	0.004*
Strongly agree	-	0.992
Social influence (SI)		
Disagree (<8 score)	Ref	
Agree (≥8 score)	19.25 (7.04–52.64)	0.0001*

* *p*-value significant.

**Table 5:** Logistic regression analysis for the effort expectancy (EE) items that could influence the intention to use telehealth.

EE items	Intention to use telehealth	
	Adjusted OR	*p*-value
Telehealth will be flexible to interact with.		
Strongly disagree	Ref	
Disagree	0.39 (0.1–10.31)	0.572
Neutral	1.17 (0.11–12.54)	0.896
Agree	5.28 (0.46–61.11)	0.183
Strongly agree	8.45 (0.55–128.84)	0.125
Learning to operate the telehealth equipment will be easy for me.		
Strongly disagree	Ref	
Disagree	50.02 (0.89–2,821.85)	0.057
Neutral	7.58 (0.38–151.85)	0.185
Agree	2.03 (0.112–36.83)	0.631
Strongly agree	0.89 (0.04–18.20)	0.941
It will be easy for me to fix the telehealth technical issues.		
Strongly disagree	Ref	
Disagree	0.71 (0.11–4.60)	0.721
Neutral	1.50 (0.26–8.65)	0.649
Agree	5.83 (0.70–48.80)	0.104
Strongly agree	2.44 (0.30–19.73)	0.401
I will find telehealth easy to use.		
Strongly disagree	Ref	
Disagree	0.14 (0.004–5.57)	0.298
Neutral	0.67 (0.03–17.32)	0.809
Agree	0.70 (0.03–17.71)	0.829
Strongly agree	2.17 (0.05–98.73)	0.690
It will be easy for me to become skillful in using telehealth.		
Strongly disagree	Ref	
Disagree	1.63 (0.03–101.45)	0.816
Neutral	6.53 (0.47–91.96)	0.165
Agree	18.52 (1.07–320.56)	0.045*
Strongly agree	30.75 (1.14–832.26)	0.042*
Using telehealth will be simple.		
Strongly disagree	Ref	
Disagree	6.00 (0.08–445.72)	0.415
Neutral	3.79 (0.20–70.82)	0.373
Agree	1.43 (0.08–25.82)	0.810
Strongly agree	0.73 (0.03–17.82)	0.850
Effort expectancy (EE)		
Disagree (≤15 score)	Ref	
Agree (>15 score)	1.92 (0.45–8.15)	0.377

* *p*-value significant.

**Table 6:** Logistic regression analysis for the facilitating condition (FC) items that could influence the intention to use telehealth.

FC items	Intention to use telehealth	
	Adjusted OR	*p*-value
I have the resources necessary to use telehealth (e.g., Computer with camera and headphone, smartphone, Internet).		
Strongly disagree	Ref	
Disagree	0.36 (0.04–3.02)	0.347
Neutral	0.91 (0.19–7.66)	0.931
Agree	0.37 (0.05–2.64)	0.319
Strongly agree	0.40 (0.05–2.99)	0.370
I have the knowledge necessary to use telehealth.		
Strongly disagree	Ref	
Disagree	2.45 (0.35–17.13)	0.368
Neutral	7.33 (1.45–37.15)	0.016*
Agree	8.70 (1.71–44.10)	0.009*
Strongly agree	5.35 (0.66–43.10)	0.115
Telehealth is compatible with other operation systems I use (e.g., Windows, Mac, Android, or IOS).		
Strongly disagree	Ref	
Disagree	0.78 (0.68–9.12)	0.847
Neutral	0.94 (0.13–6.74)	0.954
Agree	3.70 (0.47–29.44)	0.216
Strongly agree	5.19 (0.37–73.53)	0.223
Technical support is available for assistance with telehealth difficulties.		
Strongly disagree	Ref	
Disagree	3.92 (0.61–25.02)	0.149
Neutral	4.81 (1.20–19.23)	0.026*
Agree	8.65 (1.65–45.28)	0.011*
Strongly agree	20.65 (1.37–312.16)	0.029*
Facilitating conditions (FC)		
Disagree (≤10 score)	Ref	
Agree (>10 score)	2.56 (0.75–8.74)	0.133

* *p*-value significant.

## 4 Discussion

This study assessed telehealth acceptance among healthcare practitioners in the Kingdom of Saudi Arabia using a theory-based instrument grounded on the UTAUT model. The selection of the research instrument was appropriate for the context of using telehealth in the KSA and it was consistent with local validation work on a Saudi telehealth acceptance scale derived from the UTAUT (55). The analysis revealed that telehealth was highly accepted by HCPs in the KSA. Most of the HCPs in this study (97.8%) were positive about using telehealth. The high positive perception of telehealth is consistent with findings from another study that measured telehealth awareness and acceptance among HCPs in the KSA (31). Alharbi (2023) reported that awareness of telehealth was high among HCPs (91%). Specifically, 82% of HCPs from their study believed that telehealth was beneficial to them, and 84% believed it was beneficial to patients. It is important to note that Alharbi's study was conducted at the end of 2020, when telehealth usage in the KSA was in the early implementation phase. A previous study on telehealth acceptance in the KSA have reported favorable attitudes but lower specific agreement on telehealth benefit (43%) (57), likely reflecting narrower settings, earlier implementation phases, and limited sample size.

In line with previous studies that measured telehealth acceptance among HCPs, performance expectancy (PE) was a significant predictor of telehealth acceptance in our study (21–24, 29, 30). Thus, HCPs are likely to utilize telehealth if they expect benefits from its usage. As per the findings of the study, the potential benefits of using telehealth included the ability to accomplish tasks more quickly, save time, and improve the quality of care. These benefits were also reported in other studies conducted in the KSA (32, 33). However, the rates of agreement on the potential benefits of telehealth were lower than those reported in our study. In those studies, 36% to 72% of the participants believed in the potential benefits of telehealth, such as improving the quality of the healthcare system, enhancing the effectiveness of care, and saving patients' time and money. However, those studies were conducted in a limited number of hospitals across two cities with small sample sizes, which restricts the generalizability of their results. Our findings indicated that performance expectancy independently predicted the positive intention to use telehealth, indicating that clinicians are moved by tangible values including faster task completion, time saved, and preserved or improved quality of care. This pattern aligns with conclusions from international clinician samples where perceived usefulness remains the primary determinant of telehealth uptake, including multi-setting studies in Ethiopia (21), Germany (29), and Chile (58). Together, these studies reinforce a simple message: when telehealth clearly helps clinicians do their work, intention to use it follows.

The findings of this study revealed that social influence (SI) was a significant predictor of HCPs' intention to use telehealth in the KSA. This aligns with the results of several previous studies (21, 29, 58), though some studies found no such effect (30, 59). In the Saudi context, these findings are aligned with the known cultural norms. The growing of national digital health initiatives during and following the COVID-19 pandemic served to normalize virtual care services. This normalization was facilitated by high-level endorsements and peer influence, which subsequently intensified normative pressure within clinical teams. The experiences of the HCPs in the KSA with the 937 virtual medical call centers illustrate how organizational endorsement and visibility can shape HCPs acceptance toward telehealth (26, 60). Our results suggests that HCPs in the KSA are highly influenced by the opinions and behaviors of their peers when considering the adoption of telehealth. This emphasizes the importance of leadership and peer support in promoting telehealth utilization. Management teams can play a critical role by encouraging experienced users to serve as role models and ambassadors for telehealth within their clinical environments.

By contrast, effort expectancy (EE) facilitating conditions (FC) were statistically insignificant predictors of telehealth acceptance, contradicting previous research that identified EE and FC as key factors (21–24, 29, 30). Our results indicated that HCPs in the KSA may perceive telehealth as technically challenging, particularly in terms of resolving technical issues. This perceived complexity could act as a barrier to adoption, as HCPs are less likely to engage with technologies that they find difficult to operate or learn. Moreover, the participants expressed concerns about the availability of timely technical support and access to telehealth-related resources. This pattern aligns with comparative studies indicating that institutional factors such as local protocols, incentive structures, medico-legal frameworks, and IT infrastructure, can significantly influence HCPs' acceptance of telehealth (29). A key practical implication is that organizational alignment acts as a critical gatekeeping factor for successful implementation, even when individual attitudes are highly favorable. Addressing these logistical barriers, such as by providing real-time IT assistance via chat or phone, may improve acceptance levels and ease implementation efforts. Two practical interpretations are possible for our findings. First, once value and social norms are strong, ease-of-use and resource availability become less decisive for telehealth acceptance. Second, HCPs still report friction points in telehealth adoption, particularly concerning troubleshooting, workflow integration, and a lack of clear protocols. While telehealth was generally accepted, some factors limit its utilization as informational support and telehealth integration methods (52). These barriers were also reported in a study conducted in the KSA to assess utilization of the 937 virtual services (60).

This study offers several practical implications for healthcare decision-makers planning for telehealth implementation. First, highlighting the clinical and operational benefits of telehealth may increase its perceived usefulness among HCPs, particularly for those who adopt technology only when its value is evident. Second, the significant role of social influence points to the importance of showcasing successful telehealth users within the organizations to foster peer-driven acceptance. To address concerns related to effort expectancy, telehealth developers should prioritize user-friendly designs and be accompanied by hands-on training workshops to show that using telehealth is an easy way to provide healthcare services, especially for certain disciplines. Moreover, the primary objective for telehealth designers should be to enhance usability, thereby promoting greater user acceptance and adoption. Furthermore, given the concerns related to facilitating conditions, accessible and responsive IT support should be established, including real-time troubleshooting via chat or phone. The empirical findings of this study offer healthcare organizations a strategic foundation for developing interventions to address HCPs concerns regarding telehealth. Factors identified in this study can from the foundation for telehealth training programs, potential improving telehealth acceptance among HCPs.

The findings of this study offer significant theoretical and practical implications for future research related to telehealth adoption in the KSA. First, our findings offer a significant theoretical contribution to the literature relevant to applying the UTAUT model to understand telehealth acceptance. The results of this study validate the applicability of the UTAUT's constructs for explaining the HCPs' acceptance of telehealth. To the best of our knowledge, this is the first study to apply the constructs of the UTAUT to understand telehealth acceptance among HCPs in the KSA. Given the raising demand for telehealth, identifying the factors influencing its acceptance is essential for the successful implementation of new programs. Second, by applying the UTAUT model, this study establishes a foundation for future research to include additional constructs or sociodemographic factors to further improve the understanding of telehealth acceptance. A critical direction for future research is to recruit a larger sample size that is representative of HCPs across all healthcare disciplines in the KSA. A valuable extension of this study would be to integrate culturally specific constructs and key sociodemographic moderators, such as income level and geographical distance from healthcare workplace to the existing model. Incorporating more financial and social moderators could enhance the power of the model in predicting telehealth acceptance among HCPs in the KSA. To advance telehealth research activities in the KSA, the research supporting entities should encourage more researcher participation in this field and provide funding support.

This study has several limitations that should be acknowledged. The study relied on self-reported data, which may introduce response bias and may not accurately reflect actual behavior of using telehealth. Additionally, the cross-sectional design captures the intention at a single time point, which may not reflect evolving attitudes with increased exposure or system improvements. The study's methodological approach inhibits causal inferences and the examination of telehealth acceptance over time. Future research should consider longitudinal designs to track changes in acceptance over time and determine actual usage behavior. Adding a qualitative component would provide deeper insights into the underlying reasons for the lack of significance in EE and FC constructs and reveal discipline-specific barriers. Moreover, the online data collection method limited participation to HCPs with internet access and a degree of technological engagement, potentially excluding key perspectives. The use of convenience sampling may also limit the generalizability of our findings.

## 5 Conclusion

This is the first large-scale, theory-based study to assess telehealth acceptance among HCPs across all regions of the KSA. Applying the UTAUT framework, the study explored the influence of perceived performance, effort, social support, and facilitating conditions on behavioral intention to use telehealth. Performance expectancy (PE) and social influence (SI) emerged as statistically significant predictors of telehealth acceptance. In contrast, effort expectancy (EE), facilitating conditions (FC), and most sociodemographic variables were not significant predictors. These findings provide practical insights for healthcare leaders aiming to enhance the adoption and sustainability of telehealth programs. Future research should explore telehealth use across different specialties, incorporate longitudinal and qualitative methods, and further investigate structural and organizational barriers to telehealth acceptance. These efforts will support the design of more effective, user-centered telehealth strategies and contribute to the broader goals of healthcare modernization in the KSA.

## Data Availability

The original contributions presented in the study are included in the article/Supplementary Material, further inquiries can be directed to the corresponding author.
